# High‐Altitude Open‐Pit Coal Mining has Changed the Sulfur Cycle and Ecological Network of Plant Rhizosphere Microorganisms

**DOI:** 10.1002/ece3.71183

**Published:** 2025-04-11

**Authors:** Honglin Liu, Hengfang Wang, Junqing Sun, Tianhong Yang, Zhengxian Mo, Hao Huang, Yabo Pan

**Affiliations:** ^1^ School of Geology and Mining Engineering Xinjiang University Urumqi China; ^2^ Key Laboratory of Environmental Protection Mining for Mineral Resources at Universities of Education Department of Xinjiang Uygur Autonomous Region Urumqi China; ^3^ College of Ecology and Environment Xinjiang University Urumqi China; ^4^ Key Laboratory of Oasis Ecology of Ministry of Education Xinjiang University Urumqi China; ^5^ China Energy Xinjiang Tuokexun Energy co., Ltd. Turpan China; ^6^ School of Resources and Civil Engineering Northeastern University Shenyang China

**Keywords:** functional gene, microbial community, mining disturbance, rhizosphere effect, succession stage, sulfur cycling

## Abstract

Ecological restoration of mining sites has a considerable effect on microbial community dynamics; however, its impact on sulfur cycling is unclear. This study explored the changes in functional genes related to sulfur cycling and microbial diversity during different stages of succession following the ecological restoration of a mining site in a cold arid area. A total of three succession stages were selected—natural, secondary, and artificial. The expression of sulfur cycle‐related genes and associated microbial drivers was investigated using metagenomics and network analysis. The dominant bacteria in the secondary succession were found to be r‐strategy‐adopting *Proteobacteria* and *Cyanobacteria*. Natural succession primarily comprised *Aspergillus* and *Thermus*, whereas artificial succession comprised *Proteobacteria*, *Chlorophyta*, and *Actinobacteria*. Mining disturbances were determined to significantly reduce the abundance of sulfur‐cycling archaea. Secondary succession was primarily influenced by soil total phosphorus in the sulfur‐cycle gene network. The key bacteria and archaea involved in the sulfur cycle were found to be *Bradyrhizobium* and *Nitrosopumilus*, respectively. The abundance of *Streptomyces* was significantly higher in natural succession than in artificial or secondary succession. *Burkholderia*, which has biological control and bioremediation effects, was abundant during artificial succession. These results provide a theoretical basis for restoring the sulfur cycle and promoting a positive succession of ecosystems in mining areas.

## Graphical abstract

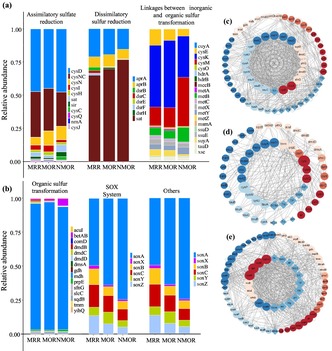

## 1 Introduction

Coal mining causes severe damage to soil ecosystems (Bian et al. 2022). Microorganisms are important indicators of soil health and are regulators of nutrient cycling, plant diseases, pests, and other processes. Clarifying the structure and function of microbial communities at different succession stages after mining disturbances and their driving mechanisms can aid in the development of effective ecosystem restoration strategies that enable positive succession (Wang et al. 2023). The sulfur (S) cycle is an important biogeochemical process that is primarily driven by microorganisms that regulate carbon and nitrogen cycling and play important roles in global climate change (Yu et al. 2021). S is an essential component of various biological molecules, including proteins, amino acids, and enzymes (He et al. 2016). It is a vital nutrient for plant growth, development, metabolism, and various important biochemical reactions, and is primarily absorbed by plants in the form of soluble sulfates (Sun and Mou 2016). Changes in carbohydrate metabolism during photosynthesis may affect plant stress resistance and respiration (He et al. 2016).

As evidenced by changes in the expression of functional genes, mining disturbances can alter soil nitrogen and phosphorus cycling by altering the physical and chemical properties of the soil. Plants require a high potential for nitrogen‐ and phosphorus‐cycling functional genes to survive under harsh environmental conditions (Wang et al. 2023). These genes help plants adapt to adversity by regulating nutrient absorption, transport, metabolism, and redistribution. Therefore, the sulfur cycle has a driving effect on the carbon and nitrogen cycles in the succession of mining areas and can also promote positive succession in mining areas. The main environmental factors that shape the soil S cycle are different due to changes in different environments; in mangrove ecosystems, nutrients (total nitrogen, total carbon, sulfur) are key drivers of the S cycle (Li et al. 2021). Analysis of acid sulfate soil showed that oxygen and pH were highly sensitive to S cycle tolerance genes (Su et al. 2017). However, the mechanisms driving the sulfur cycle and microbial succession after disturbances in cold mining areas remain unclear.

Sulfur is transferred to the water, soil, and atmosphere by microorganisms during the decomposition of dead animals and plants (Singh et al. 2020). The S cycle regulates energy exchange by changing the valence state of sulfur, involving eight key processes—assimilatory sulfate reduction, dissimilatory sulfur reduction and oxidation, sulfur reduction, SOX systems, sulfur oxidation, sulfur disproportionation, organic sulfur transformation, and linkages between inorganic and organic sulfur transformations. The key functional genes vary in each process. For example, betABC is associated with the utilization of the sulfate ester choline‐o‐sulfate (Landa et al. 2019), and the phsABC gene family encodes a sulfate reductase for the transformation of sulfate to other sulfate forms and sulfide. Changes in the functional gene abundance can alter the S cycle. However, it is still unclear how key genes and their main drivers in each process of the S cycle vary during different succession stages after a mining disturbance.

Ecological and environmental issues related to open‐pit coal mining need to be urgently addressed in cold and arid northwestern China. Vegetation succession in the mining area can improve soil structure and enhance nutrient cycling by changing the composition and function of soil microbial communities (Li et al. 2016). In particular, the activity of sulfur‐oxidizing and reducing microorganisms has a crucial impact on the sulfur cycle (Sun et al. 2023a). Therefore, the positive succession after ecological restoration promotes the development of the ecosystem in a more favorable direction (Bian et al. 2022; Coban et al. 2022), which is critical for the sustainable development of mining and the effective restoration of ecosystem services.

Based on the above, this study investigated the changes in S cycling in the soils of the Heishan coal mine, a highly cold mining area in the Xinjiang Uygur Autonomous Region, China, which is actively being restored. A total of three sites were selected representing different stages of succession—vegetation growing naturally outside the mining area (primary succession; NMOR), vegetation growing naturally inside the mining area (secondary succession; MOR), and vegetation planted during the ecological restoration of the mining area (artificial succession; MRR). The expression of sulfur cycle‐related genes and associated microbial drivers was investigated using metagenomics and network analysis. In the different stages of succession, the study aimed to (1) describe the functional genes in sulfur cycling processes and their network relationships, (2) determine changes in their abundance and key factors regulating sulfur cycling bacteria and archaea, and (3) identify key microbial species.

## 2 Materials and Methods

### 2.1 Overview of the Study Area

The Xinjiang Heishan open‐pit coal mine is located in a mountainous valley north of the middle section of the Tianshan Mountains (87°26′04″–87°29′40″ E, 43°13′48″–43°14'0 1″N). The mining area has a continental, arid, high‐altitude climate. The average annual precipitation and temperature are 215.3 mm and 1.2°C ~ 2.3°C, respectively. There are strong winds in the spring, with a maximum wind speed of 13 m/s. The vegetation coverage is low, and wind occurs year‐round. The main types of soil erosion are slight water and wind erosion (Nigati 2017). The natural vegetation types present were typical grassland vegetation, such as *Stipa capillata*, *Chenopodium glaucum* , and *Elymus nutans* (Table S1).

### 2.2 Soil Collection and Measurement

A total of three succession types were selected for this study—primary succession (naturally growing plants outside the mining area, NMOR), secondary succession (naturally growing plants in the mining area, MOR), and artificial succession (vegetation planted during ecological restoration in the mining area, MRR). In July 2021, three plots (1 × 1 m) were selected for each succession type, with each plot spaced more than 10 m apart. Three replicates were selected for each plant in the sample plot. The entire plant was excavated using a sterile shovel, and the loose soil attached to the roots was shaken off. The roots were placed in sterile bags for low‐temperature storage and brought back to the laboratory then rinsed with PBS solution (137 mmol/L NaCl, 2.7 mmol/L KCl, 8.5 mmol/L Na_2_HPO_4_, 1.5 mmol/L KH_2_PO_4_, pH 7.3). The rhizosphere soil was thoroughly cleaned (Edwards et al. 2015) and centrifuged at a low temperature at 10,000 r for 1 min to concentrate the sample. The sample was then stored at a low temperature for further use in microbiological experiments. A total of 33 samples were used for metagenomic sequencing. The other soil was used to measure the physical and chemical properties of the soil, which was naturally dried, ground, and sieved. Aluminum boxes were used to collect the root soil to measure the soil moisture content. The methods for measuring the soil indicators are listed in Table 1 (Bao 2000).

**TABLE 1:** Main soil indicators and their measurement methods.

Index	Measurement method
pH	In a 5:1 water: soil suspension
Soil electrical conductivity (EC, Cond)	The oscillation method
Soil moisture content (SWC, %)	Fresh soil samples were dried at 105°C to a constant weight
Soil organic matter content (SOM)	The potassium dichromate‐concentrated sulfuric acid external heating method
Soil total phosphorus (TP)	The NaOH fusion molybdenum‐antimony anti‐colorimetric method
Soil‐available phosphorus (AP)	The sodium bicarbonate leaching method and molybdenum antimony anti‐colorimetric method
Soil total nitrogen (TN)	Potassium dichromate and the Kjeldahl method
Soil nitrate‐nitrogen (NO_3_‐N)	The phenol disulfonic acid colorimetric method
Soil ammonium‐nitrogen (NH4 + ‐N)	The indophenol blue colorimetric method
Soil total potassium (TK) and available Potassium (AK)	A flame photometer
Total soil mercury content (Hg)	Atomic fluorescence

### 2.3 DNA Extraction and Sequencing

The total genomic soil DNA was extracted from the rhizosphere/bulk soil using a Power Soil DNA isolation kit (Omega, Bio‐Tek, Norcross, GA, USA) following the manufacturer's instructions, and the quality of the DNA was assessed using a NanoDrop ND‐2000 spectrophotometer (Thermo Fisher Scientific, Wilmington, NC, USA). Metagenomic sequencing was performed by Sangon Biotech Company (Shanghai, China) on an Illumina NovaSeq platform (PE 1500) at a sequencing depth of 6 GB/sample. All the raw data were submitted to the NCBI for the Biotechnology Information Sequence Read Archive database (accession number PRJNA860834).

### 2.4 Metagenomic Assembly

Quality adjustments were made to the original reads by removing adapters, reads < 35 nucleotides in length, and low‐quality reads (Q < 20) using the Trimmomatic software (version 0.36). High‐quality paired‐end reads were assembled into overlapping groups. Prodigal (version 2.60) was used to evaluate the quality of overlapping populations and predict the S cycle gene function (Liu et al. 2013). DIAMOND software (version 2.0) and BLASTP (BLAST version 2.2.28+) were used to search for predicted genes with the NCBI nonredundant protein database (E‐value cutoff = 10^−5^) (Altschul et al. 1997). Functional annotations were completed using KOBAS 2.0 (KEGG orthography‐based annotation system) to align the reads with the KEGG data (Xie et al. 2011).

### 2.5 Analysis of Processes Related to the Sulfur Cycle

The association of the sulfur cycle was analyzed using six cyclic pathways—assimilation sulfate reduction (ASR), dissimilatory sulfur reduction and oxidation (DSR), sulfur oxidation (SO), sulfur disproportionation (SD), organic sulfur transformation (OST), and linkages between inorganic and organic sulfur transformation (LBIOST), SOX systems, and others (Yu et al. 2021). These processes mediate the absorption and transportation of sulfur and directly affect soil sulfur levels. The detailed functional classifications are presented in Table S2.

### 2.6 Statistical Analysis

Shapiro–Wilk and Levene's tests were used to assess data normality and homogeneity using the multicomp package in R software (version 3.5.2) (Hothorn et al. 2008). Significant differences in the soil physicochemical properties and the relative abundance of functional genes in the three succession stages were analyzed using Fisher's least significant difference (LSD) test (*p* < 0.05). The similarities between the sulfur‐cycling microbial communities were explored at different succession stages using the vegan package (version 2.5–6) (Oksanen et al. 2019) for similarity analysis (ANOSIM). The ggplot2 package (version 2.2.0) (Wickham 2016) was used for principal coordinate analysis (PCoA) based on weighted UniFrac distances. The ggraph package (version 2.1.0) (Pedersen 2022) was used to generate the graphs. Random matrix theory was used to construct microbial interaction networks. The Random Matrix Theory (RMT) identifies non‐random biological correlations by comparing the eigenvalue distributions of actual matrices with those of random matrices and then constructs a Molecular Ecological Network (MEN) that features automatic definition and robustness against noise (Deng et al. 2012). The thresholds were determined based on the Spearman correlation matrix and random matrix theory to construct sulfur‐cycling gene networks for the three succession stages. The relative abundance of the dominant bacteria, archaea, and S‐cycle genes was first calculated and was followed by the use of the Spearman correlation coefficient to determine their interaction and construct a symmetric correlation matrix. The same method was used to analyze the correlation between environmental factors, microorganisms, and functional genes. The interaction networks were visualized using Cytoscape 3.6.1. The Mantel test was used to analyze the optimal subset of environmental variables affecting the microbial community structure and functional gene abundance.

## 3 Results

### 3.1 Physical and Chemical Attributes of the Soil Factors

**FIGURE 1:**
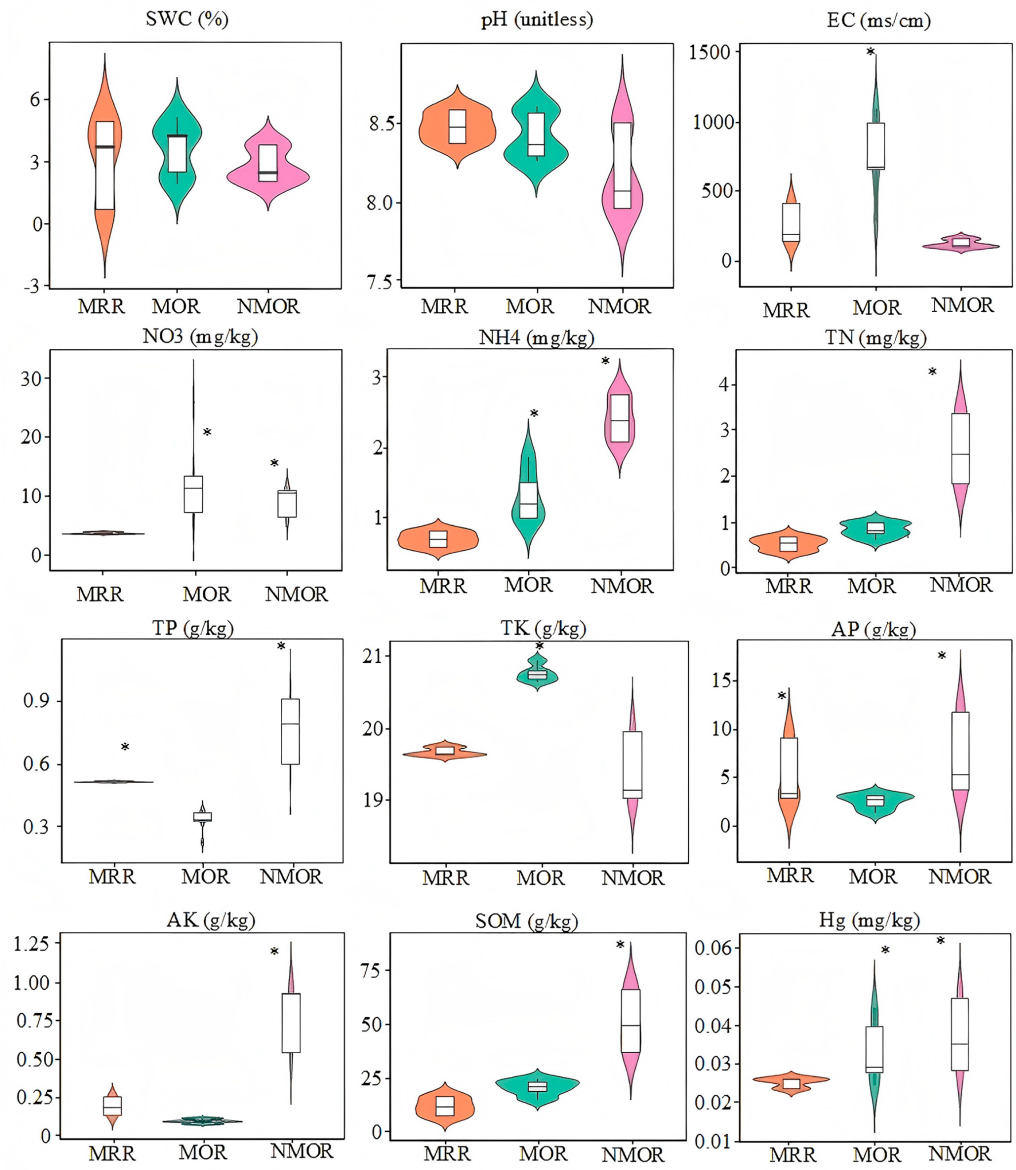
Physical and chemical properties of rhizosphere soil in the different succession stages.

As per the soil physicochemical factor analysis (Figure 1 and Table S3), the electrical conductivity and total potassium content of the rhizosphere soil of the NMOR plants were significantly higher than those of the rhizosphere soils of the MOR and MRR plants. The nitrate‐nitrogen, ammonium‐nitrogen, and mercury contents in the rhizosphere soils of the NMOR and MOR plants were significantly higher than those of the MRR plants. The total and available phosphorus were significantly higher in the MRR and NMOR rhizosphere soils than in the MOR soil. The total nitrogen, available potassium, and organic matter content in the rhizosphere soil of NMOR plants were significantly higher than those in the MOR and MRR soils. There were, however, no significant differences in the soil moisture content or pH values among the three groups.

### 3.2 Differences in Key Functional Genes of the S Cycle Between Succession Stages

**FIGURE 2:**
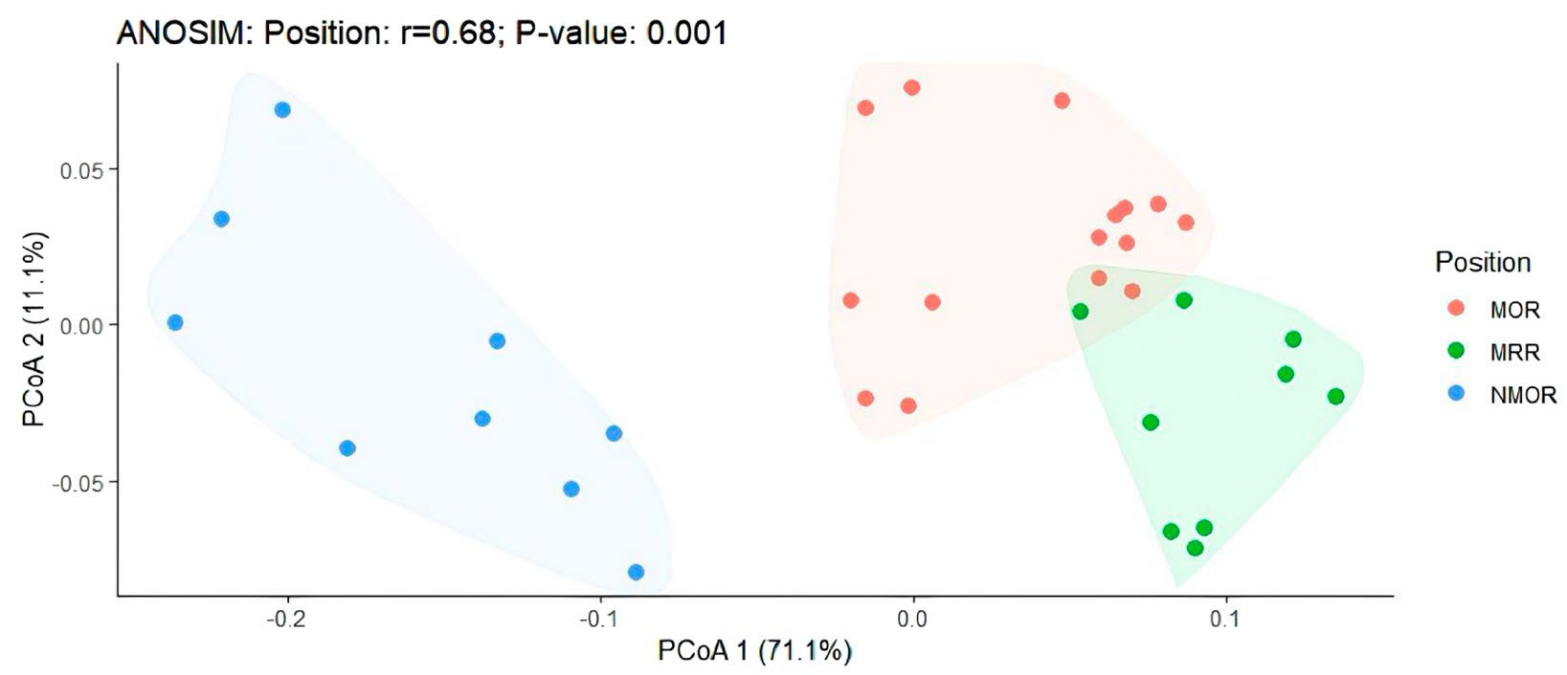
PCoA of microbial composition related to sulfur cycling in rhizosphere soils of different succession types.

The composition of the microbial genes involved in the sulfur cycle varied among the succession stages (*p* = 0.001) (Figure 2). The different succession stages explained 68% of the changes in the sulfur cycle, with the first PCoA axis accounting for 71.1% of the total explained amount, the second axis accounting for 11.1%, and both axes accounting for 82.2%. These results indicate that the succession stage is the main factor influencing sulfur cycling.

**FIGURE 3:**
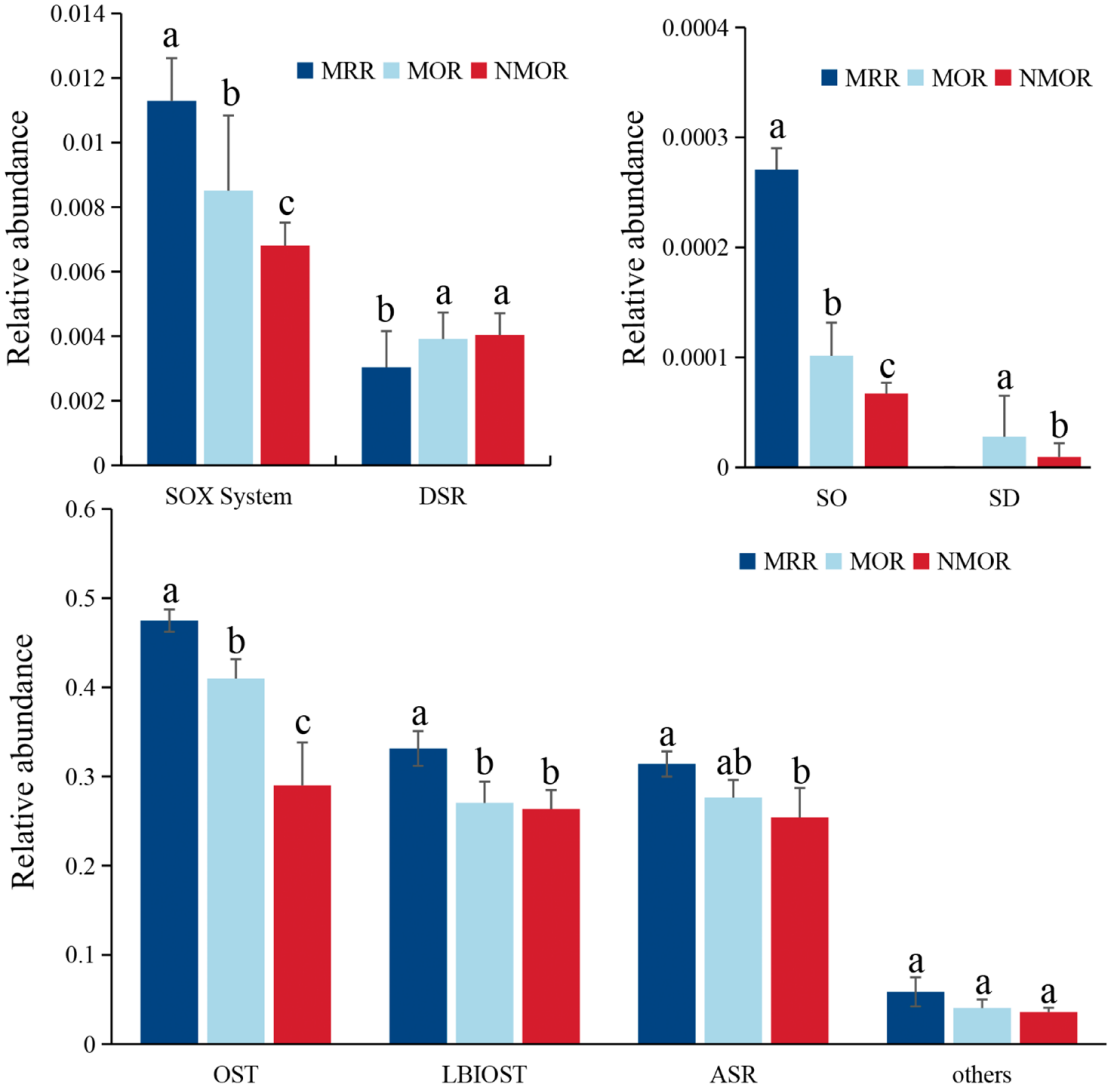
Relative abundance of sulfur cycle‐related genes in different succession stages.

By comparing the proportions of different functional genes involved in the sulfur cycle, it was found that the highest gene abundance was associated with OST, followed by LBIOST and ASR (Figure 3). The SOX systems and DSR systems were one order of magnitude lower than those of the previous systems.

Differential analysis of the functional genes involved in the eight processes of sulfur cycling at different succession stages demonstrated that MRR>MOR>NMOR in 6 cycles of the SOX system, SO, OST, LBIOST, ASR, and others. There was no significant difference in LBIOST between MOR and NMOR, unlike the other processes. DSR exhibited an opposite trend, whereas MRR did not contain any SD‐related genes. The relative abundances of genes in the SO and SD systems were relatively low; only glpE and sqr were observed in the SO system, whereas only phsC was observed in the SD system.

**FIGURE 4:**
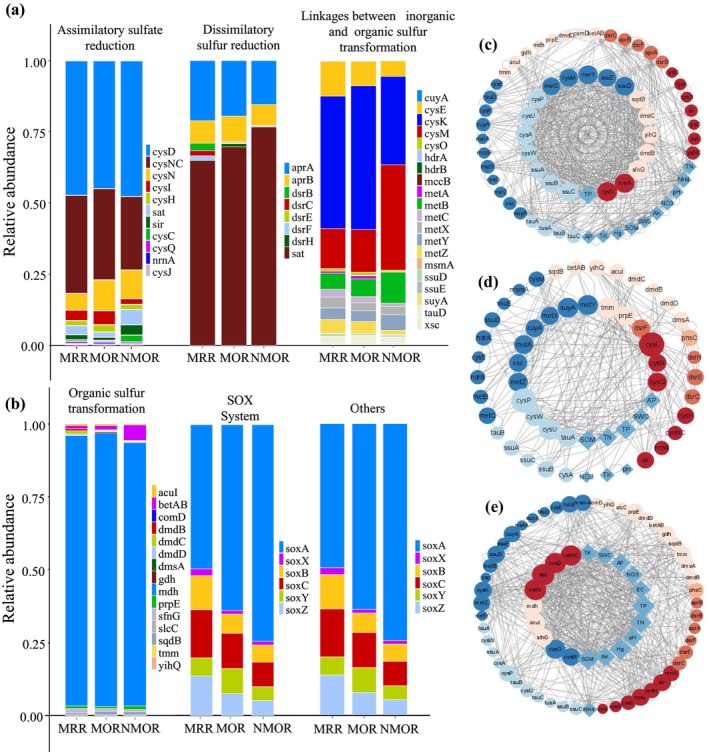
Relative abundance and network characteristics of sulfur cycle‐related genes in different succession stages: (a, b) Show stacked diagrams of the relative gene abundance under each of the eight processes of sulfur cycling; (c–e) show the functional gene network diagrams for MRR, MOR, and NMOR, respectively.

In the ASR process, cysD, cysNC, and cysN genes dominated the three succession stages (Figure 4a). The relative abundance of cysI was higher in the MOR than in the MRR and NMOR, whereas the relative abundances of sat and sir were NMOR>MRR>MOR. The number of gene types was significantly higher in MRR than in MOR and NMOR, and the genes varied at different stages of succession. In the OST process (Figure 4b), mdh was dominant and betAB was NMOR>MOR>MRR. In the SOX System, soxA exhibited the order NMOR>MOR>MRR, whereas soxC, soxZ, and soxB exhibited the opposite trend. Among the other genes, cysA and cysW were MOR>MRR>NMOR.

By sorting the network parameters, the key functional genes and environmental factors affecting the S cycle (Figure 4c–e) were selected and analyzed as their key drivers in the network. MRR and NMOR were more complex than the MOR. Network complexity was primarily affected by assimilatory sulfur reduction, linkages between inorganic and organic sulfur transformations, organic sulfur transformations, and other processes in the MRR; assimilatory sulfur reduction and linkages between inorganic and organic sulfur transformations in the MOR; and assimilatory sulfur reduction, linkages between inorganic and organic sulfur transformations, and organic sulfur transformations in the NMOR.

TP was the only key environmental factor affecting the S cycle in the MRR, while several soil factors participated in MOR and NMOR, such as AP, SWC, TP, TN, and SOM. Soil factors participating in the NMOR S cycle included TK, SWC, AP, NO3, EC, TP, TN, PH, Hg, AK, and SOM.

### 3.3 Main Microbial Lineages Involved in the S Cycle

Based on the abundance of the main microorganisms involved in the S cycle (Table S2), it was found that the most abundant bacteria primarily participated in the SOX system, assimilatory sulfate reduction, and organic sulfur transformation. The most abundant archaea participated in dissimilatory sulfur reduction and oxidation, sulfur oxidation, sulfur reduction, organic sulfur transformation, linkages between inorganic and organic sulfur transformations, and other processes.

**FIGURE 5:**
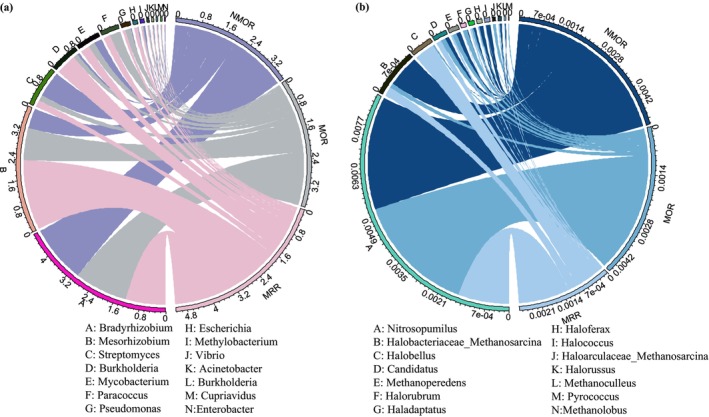
Sulfur cycle‐related microorganisms and relative abundance drive different succession stages: (a) Bacteria and (b) Archaea.

The main bacteria involved in the sulfur reduction were determined to be *Bradyrhizobium*, *Pseudomonas* (phylum *Proteobacteria*), *Mesorhizobium* (*Planctomycetes*), and *Streptomyces* (phylum Cyanobacteria; Figure 5a and Table S2). The main archaea participating in the S cycle were *Nitrosopumilus* (phylum *Thaumarchaeota*), *Halobacteriaceae* (*Crenarchaeota*), *Methanosarcina* (*Euryarchaeota*), *Halobellus* (*Candidatus Korarchaeota*), and *Candidatus* (*Candidatus Bathyarchaeota*; Figure 5b and Table S2).

**FIGURE 6:**
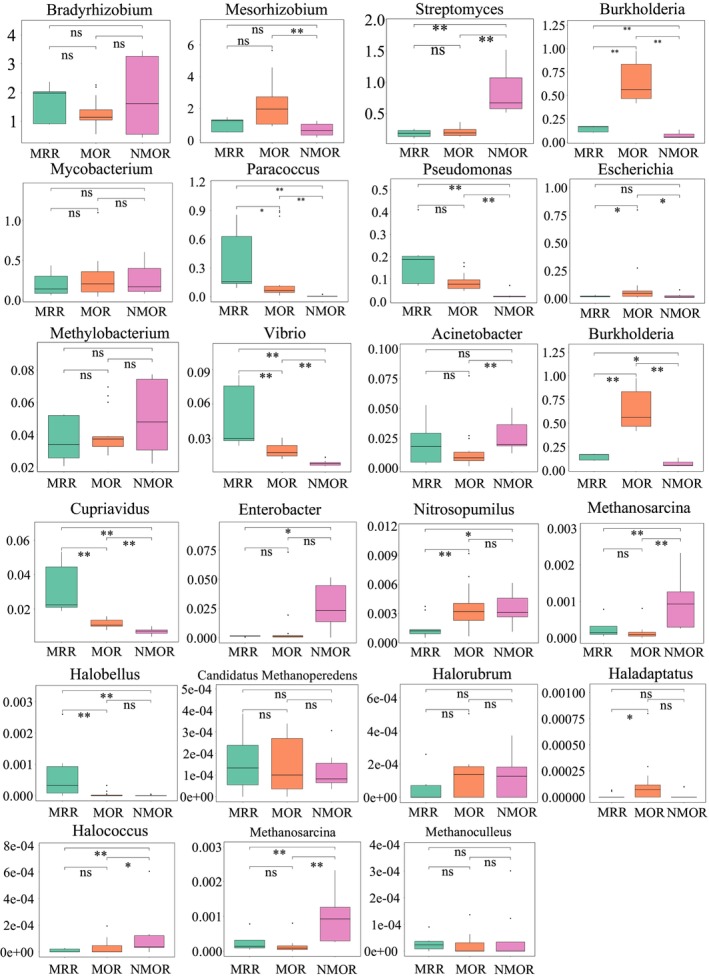
Key microbial species involved in the sulfur reduction and oxidation processes in the different succession stages.

The relative abundance of bacteria varied among the different succession stages (Figure 6). *Streptomyces* (*Cyanobacteria*) were observed to be more abundant in the NMOR than in the MOR and MRR, whereas there was no significant difference observed between the MRR and MOR. The abundance of *Paracoccus* (*Chlorobi*), *Burkholderia*, *Vibrio* (*Firmicutes*), and *Cupriavidus* (*Aquificae*) varied significantly between succession stages.

The relative abundance of archaea at different succession stages (Figure 6) was analyzed, and it was found that *Nitrosopumilus* (*Thaumarchaeota*) was significantly more abundant in the NMOR and MOR than in the MRR, and *Halococcus* was significantly more abundant in the NMOR than in the MOR and MRR.

## 4 Discussion

### 4.1 Differences in S Cycle Characteristics Among Different Succession Stages

The soil–plant system is an important component of the sulfur biogeochemical cycle. This study demonstrated significant differences in the soil physicochemical properties and microbial gene composition at the three successional stages (Figure 2). This is primarily because soil sulfur is divided into organic and inorganic sulfur, with their proportions varying with soil type, plant species, water status, microbial activity, environmental conditions, and organic matter content (Vandenkoornhuyse et al. 2003; Ahmad et al. 2016; van Veelen et al. 2020; Ma et al. 2022).

Strong relationships were observed between organic sulfur conversion, inorganic sulfur, and organic sulfur, and the relative abundance of assimilatory sulfate reduction genes (Figure 3), indicating that these processes play a vital role in the S cycle (Koch and Dahl 2018; Yu et al. 2021). Gene abundance was greater in artificial succession than in secondary and primary succession, which is inconsistent with the N and P soil cycles (Wang et al. 2023). This indicated that the S cycle is more active in highly variable environments (Hausmann et al. 2018).

In contrast to the results of this study, previous studies have demonstrated that the relative abundance of SOX system functional genes is higher than that in other processes. This is likely because the previous studies were mostly based on natural ecosystems, whereas the present study focused on herb‐dominated ecosystems affected by mining disturbances (Yu et al. 2021; Li et al. 2022).

Assimilatory sulfate reduction, organic sulfur transformation, and linkages between inorganic and organic sulfur transformations are the three dominant succession processes. The dissimilatory sulfur reduction and oxidation activities were generally low, which is consistent with the results of Li et al. (2022) and Vikram et al. (2016). dsrB is an indicator of the diversity and composition of sulfite‐/sulfate‐reducing microorganisms. This was detected only in the MRR in this study, indicating that dissimilatory sulfur reduction and oxidation were widely present in highly variable environments during artificial succession. The sat gene was enriched in the DSR process and was primarily involved in the transformation of sulfate and APS, with its expression in the order NMOR>MOR>MRR, indicating the active degree of sulfate and APS transformation. Simultaneously, aprAB participated in APS and sulfate conversion, and its expression followed the order MRR>MOR>NMOR. These results are consistent with the relatively high APS and sulfate conversion activities in variable environments (Yu et al. 2021).

The concentration of S can alter plant functional traits, such as the ratio of the main and lateral roots and the development of root hair (Ahmad et al. 2016). It was found that the abundance of genes associated with the S cycle was significantly higher in the MRR than in the MOR, which is also related to the characteristics of plants at different succession stages. The plants in the MRR demonstrated well‐developed lateral and fibrous roots with strong ground‐grasping ability. Low concentrations of S in the MOR and NMOR, however, inhibit the development of lateral roots (Dan et al. 2007). Additionally, the abundance of soil impurities, such as stones in the MRR, also promotes plant ground‐grasping ability, thereby promoting S cycling and increasing the S supply (Li et al. 2022).

### 4.2 Functional Genes of the S‐Cycle and Their Interactions in Different Succession Stages

The natural sulfur cycle is highly complex. The symbiotic patterns of microbial communities change under changing environmental conditions, which, in turn, affect soil function and stability (Coyte et al. 2015). It was found that the complexity of the sulfur cycle microbial network in the MOR was lower than that in the NMOR and MRR, indicating significant differences in the interactions between sulfur cycle‐related microbes at different stages of succession.

The artificial succession stage exhibited the greatest complexity, whereas the secondary succession stage, which had the harshest soil conditions, exhibited the lowest complexity. Positive and negative connections are important features of the mechanistic networks of microbial communities (Zhou et al. 2020; Li et al. 2022). Positive interactions dominated all the network diagrams, indicating that microorganisms were primarily synergistic (Hoek et al. 2016), whereas microorganisms in the MOR exhibited negative interactions with environmental factors, indicating intense resource competition (Table S2). In the MOR, soil factors were not the main limiting factors. This indicated that resource competition was relatively low during the early stages of succession and that plants could meet their growth requirements and reproduce. On the other hand, soil factors were the limiting factors in the NMOR and competition was the main mechanism for coexistence. The low network complexity of the MOR also indicates that secondary succession is slow. However, artificial restoration can significantly accelerate the recovery of ecosystems through measures such as vegetation reconstruction and soil improvement (Kuerban et al. 2024). Therefore, appropriate artificial restoration can promote community succession.

Total nitrogen and phosphorus are important factors affecting functional genes of the sulfur cycle in the MOR and NMOR (Zhu et al. 2018; Buongiorno et al. 2019), which also reflects the association between sulfur cycling and nitrogen and phosphorus cycling in natural ecosystems. Total nitrogen was, however, not the main influencing factor in the MRR. This indicates that the artificial restoration practices disrupted the original ecological balance and decoupled the nitrogen cycle. For example, Tian et al. (2023) found that the restoration of plantations enhanced the potential of microorganisms to fix N and denitrification processes, thus changing the balance of the nitrogen cycle.

### 4.3 Key Microbes Involved in the S Cycle

The process of degraded ecosystems restoration is a process of community succession, and changes in the quantity and quality of plant inputs at different successional stages can lead to changes in soil microbial biomass, activity, and community structure (Smith et al. 2015). The study showed that soil succession stages and microbial patterns have positive effects on plant communities (Ren et al. 2019). Environmental heterogeneity at different successional stages may lead to significant differences in microbial succession patterns (Liu et al. 2020). There are differences in the dominance of bacteria and fungi in the early and late stages of succession. Bacteria switch from r‐strategy to k‐strategy with the progress of succession, while fungi show the opposite mechanism (Zhou et al. 2017). Therefore, the relative abundance of bacterial and fungal communities at different succession stages will be different.

It was found that the microorganisms involved in the sulfur cycle primarily included bacteria and archaea, with the dominant bacteria being *Proteobacteria*, *Actinobacteria*, *Planctomycetes*, *Thermococcus*, and *Firmicutes*. Consistent with previous results (Zhang et al. 2021), sulfur‐oxidizing bacteria oxidized low‐valent sulfur elements to sulfates under aerobic or anaerobic light conditions, and sulfate was reduced to sulfide by sulfate‐reducing bacteria under anaerobic conditions. In addition, bacteria could also oxidize reduced sulfur, which may transform metal(loid)s (Sun et al. 2024a) or promote nutrient conditions (Sun et al. 2024b) in mining regions. *Proteobacteria* are a common bacterial group in mining‐disturbed soils primarily because of their capacity to grow rapidly in extremely unstable soils (Goldfarb et al. 2011; Zeng et al. 2017), thereby maintaining normal soil metabolism in extreme environments. Moreover, an increase in easily decomposable organic matter (such as glucose and root exudates) in the soil stimulates a rapid increase in r‐strategy bacteria, which establishes positive feedback for the decomposition of soil organic matter (Morrissey et al. 2017; Bernard et al. 2007).

Archaea are important indicators of community succession after environmental interference. The dominant archaea participating in the sulfur cycle were *Thaumarchaeota*, *Crenarchaeota*, and *Guangarchaea*. *Thaumarchaeota* and *Crenarchaeota* also play significant roles in N and P cycles during the succession of mining areas (Wang et al. 2023). *Thaumarchaeota* can obtain ammonia from urea and cyanate salts and participate in the N cycle, and their core chemical autotrophic metabolism can adapt to various environments (Baker et al. 2020). *Crenarchaeota* play an important role in extreme environments such as high temperatures and dry heat. *Crenarchaeota* are commonly considered the dominant aerobic soil archaea (Timonen and Bomberg 2009) and play an important role in regulating soil pH. *Crenarchaeota* are commonly found in farmlands (Bintrim et al. 1997; Simon et al. 2000), grasslands (Nicol et al. 2003a, 2003b), and degraded high mountain soils (Nicol et al. 2006), and play a vital role in soil nitrogen, carbon, and sulfur cycling (Nicol et al. 2005). Archaea have traditionally been referred to as methanogens. Some archaea also participate in anaerobic methane oxidation and hydrocarbon degradation. Archaea typically participate in the cycling of sulfur, nitrogen, and iron (Baker et al. 2020). For example, the newly described taxa *Hadesarchaea* and *Theionarchaea* possess nitrite‐ and sulfur‐reducing abilities. Changes in microbial communities during succession, therefore, depend on the nutrient content and initial microbial heterogeneity.

*Bradyrhizobium* is a nitrogen‐fixing bacterium that coexists with nitrogen‐fixing plants and relies on the close relationship between nitrogen and sulfur cycling (Davidian and Kopriva 2010). *Streptomyces* is the largest and most representative branch of cyanobacteria and is rich in antibiotics (Dong et al. 2023); plant disease resistance is, consequently, greater in the NMOR than in the MOR and MRR. *Paracoccus* is the dominant bacterial species involved in denitrification. Studies have demonstrated that *Paracoccus* increases with plant community succession (Sun et al. 2023b), which is contrary to the results of this study. *Paracoccus* is a non‐halophilic bacterial species (Zhu et al. 2020; Al‐Shayeb et al. 2021) and was, therefore, more abundant in the soil of the secondary and artificial successions, which were turned over and had a lower salt content than that in the soils subjected to the natural succession, which had a higher salinity. *Burkholderia* species are used for biological control, plant growth, and bioremediation. It demonstrated the highest abundance in the artificial succession, primarily because of its ability to produce various antibacterial metabolites to cope with complex and dynamic environments. This indicates that ecological restoration can improve the ecological function of soils in mining areas. *Vibrio* species typically inhibit the metabolism of glucose and amino acids in plants and animals (Zhao et al. 2023). It was found that the abundance of *Vibrio* was higher in secondary succession than in artificial and natural succession, indicating that the risk of *Vibrio* infection was lower in plants under artificial succession. *Cupriavidus* is a genus of denitrifying bacteria that undergoes significant changes with habitat, season, and plant succession and is also significantly correlated with soil nitrogen concentrations.

Key genes in the sulfur cycle are also related to the nitrogen cycle. *Nitrosopumilus* was the dominant archaeal species, whereas *Methanoperedens* is a methane‐eating *Candidatus Bathyarchaeota* strain that increases the metabolic rate of organisms. Metabolic activity is, therefore, higher in natural succession than in secondary or artificial succession. *Halococcus* is a halophilic archaeon that dominates high‐salinity environments (Oren 2008). Although soil conductivity was the highest in the MOR, *Halococcus* abundance was the highest in NMOR. Primary succession soils have higher stability than secondary succession soils. This suggests that the growth status of *Halococcus* improves in stable soils.

## 5 Conclusions

Significant differences were observed in sulfur cycle‐related soil microorganism communities at different succession stages in the cold and arid mining areas. The k‐strategy‐adopting *Proteobacteria* had an advantage in both natural and artificial succession, whereas the r‐strategy‐adopting *Actinomycetes* had a greater advantage in artificial succession than in primary and secondary succession. Mining disturbances significantly reduced the relative abundance of archaea, which play an important role in the microbial community structure and sulfur cycling. The overall performance of sulfur cycle‐related genes was greater in artificial succession than in secondary and natural succession. Assimilatory sulfate reduction, organic sulfur transformation, and linkages between inorganic and organic sulfur transformations were the dominant processes in the three succession stages. The physical and chemical properties of the soil had an important impact on soil succession in and outside the mining areas; the original soil in the mining area was, however, primarily limited by the total soil phosphorus content. The microbial structure of the plant rhizosphere was more stable under artificial succession than under natural or secondary succession. *Bradyrhizobium* and *Nitrosopumilus*, nitrogen‐fixing bacteria, and archaea are key microbial species in the sulfur cycle. *Burkholderia*, which has biological control and bioremediation activities, is abundant in artificial succession. These results provide a theoretical basis for ecological restoration and clarify the factors associated with positive community succession in mining‐disturbed ecosystems. This study plays an important role in promoting the positive succession of the mining ecosystem by exploring the structure and function of sulfur cycling microbial communities in different succession stages after mining disturbance, as well as their driving mechanisms. It also provides a foundation for the restoration of the ecosystem to its original state after mining disturbance. However, the research method only uses space instead of time, lacking long‐term tracking and monitoring, which is also the direction of future research.

## Author Contributions

**Honglin Liu:** conceptualization (equal), methodology (equal), supervision (equal), writing – original draft (equal). **Hengfang Wang:** data curation (equal), software (equal), visualization (equal), writing – review and editing (equal). **Junqing Sun:** investigation (equal), software (equal), supervision (equal). **Tianhong Yang:** supervision (equal). **Zhengxian Mo:** investigation (equal). **Hao Huang:** investigation (equal). **Yabo Pan:** investigation (equal).

## Conflicts of Interest

The authors declare no conflicts of interest.

## Supporting information


**Data S1.** File Legends.

File S1.

File S2.

File S3.

Data S2.

## Data Availability

All data necessary to replicate our results is available in the Data S1, File S1, File S2, File S3.
