# Supplementary material for: High‐Altitude Open‐Pit Coal Mining has Changed the Sulfur Cycle and Ecological Network of Plant Rhizosphere Microorganisms
Source: Ecol Evol. 2025 Apr 11;15(4):e71183. doi: 10.1002/ece3.71183 (PMC11992355; doi:10.1002/ece3.71183)
Supplement: Supplementary file 1 — Data S1. File Legends. [file ECE3-15-e71183-s003.docx]

**Supplementary File S1.** Physical and chemical properties of rhizosphere soil in the different succession stages.

**Supplementary File S2.** R code used to run the physical and chemical properties of rhizosphere soil in the different succession stages analyse.
