# Supplementary material for: High‐Altitude Open‐Pit Coal Mining has Changed the Sulfur Cycle and Ecological Network of Plant Rhizosphere Microorganisms
Source: Ecol Evol. 2025 Apr 11;15(4):e71183. doi: 10.1002/ece3.71183 (PMC11992355; doi:10.1002/ece3.71183)
Supplement: Supplementary file 4 — File S3. [file ECE3-15-e71183-s005.pdf]

```r
library(ggplot2)
library(ggsci)
#install.packages("hrbrthemes")
library(hrbrthemes)
library(ggplot2)
library(RColorBrewer)
library(SuppDists)




mydata<-read.csv("soil.csv")
ggplot(mydata, aes(Class, SWC))+
  geom_violin(aes(fill = Class),trim = FALSE)+
  geom_boxplot(width = 0.2)+
  scale_fill_manual(values=c(brewer.pal(7,"Set2")[c(1,2,4,5)]))+
  theme_classic()+
  theme(panel.background=element_rect(fill="white",colour="black",size=0.25),
        axis.line=element_line(colour="black",size=0.25),
        axis.title=element_text(size=13,face="plain",color="black"),
        axis.text = element_text(size=12,face="plain",color="black"),
        legend.position="none"
  )



ggplot(mydata, aes(Class, pH))+
  geom_violin(aes(fill = Class),trim = FALSE)+
  geom_boxplot(width = 0.2)+
  scale_fill_manual(values=c(brewer.pal(7,"Set2")[c(1,2,4,5)]))+
  theme_classic()+
  theme(panel.background=element_rect(fill="white",colour="black",size=0.25),
        axis.line=element_line(colour="black",size=0.25),
        axis.title=element_text(size=13,face="plain",color="black"),
        axis.text = element_text(size=12,face="plain",color="black"),
        legend.position="none"
  )


ggplot(mydata, aes(Class, EC))+
  geom_violin(aes(fill = Class),trim = FALSE)+
  geom_boxplot(width = 0.2)+
  scale_fill_manual(values=c(brewer.pal(7,"Set2")[c(1,2,4,5)]))+
  theme_classic()+
  theme(panel.background=element_rect(fill="white",colour="black",size=0.25),
        axis.line=element_line(colour="black",size=0.25),
        axis.title=element_text(size=13,face="plain",color="black"),
        axis.text = element_text(size=12,face="plain",color="black"),
        legend.position="none"
  )


ggplot(mydata, aes(Class, NO3))+
  geom_violin(aes(fill = Class),trim = FALSE)+
  geom_boxplot(width = 0.2)+
  scale_fill_manual(values=c(brewer.pal(7,"Set2")[c(1,2,4,5)]))+
  theme_classic()+
  theme(panel.background=element_rect(fill="white",colour="black",size=0.25),
        axis.line=element_line(colour="black",size=0.25),
        axis.title=element_text(size=13,face="plain",color="black"),
        axis.text = element_text(size=12,face="plain",color="black"),
        legend.position="none"
  )



ggplot(mydata, aes(Class, NH4))+
  geom_violin(aes(fill = Class),trim = FALSE)+
  geom_boxplot(width = 0.2)+
```

```
  scale_fill_manual(values=c(brewer.pal(7,"Set2")[c(1,2,4,5)]))+
  theme_classic()+
  theme(panel.background=element_rect(fill="white",colour="black",size=0.25),
        axis.line=element_line(colour="black",size=0.25),
        axis.title=element_text(size=13,face="plain",color="black"),
        axis.text = element_text(size=12,face="plain",color="black"),
        legend.position="none"
  )

ggplot(mydata, aes(Class, TN))+
  geom_violin(aes(fill = Class),trim = FALSE)+
  geom_boxplot(width = 0.2)+
  scale_fill_manual(values=c(brewer.pal(7,"Set2")[c(1,2,4,5)]))+
  theme_classic()+
  theme(panel.background=element_rect(fill="white",colour="black",size=0.25),
        axis.line=element_line(colour="black",size=0.25),
        axis.title=element_text(size=13,face="plain",color="black"),
        axis.text = element_text(size=12,face="plain",color="black"),
        legend.position="none"
  )


ggplot(mydata, aes(Class, TP))+
  geom_violin(aes(fill = Class),trim = FALSE)+
  geom_boxplot(width = 0.2)+
  scale_fill_manual(values=c(brewer.pal(7,"Set2")[c(1,2,4,5)]))+
  theme_classic()+
  theme(panel.background=element_rect(fill="white",colour="black",size=0.25),
        axis.line=element_line(colour="black",size=0.25),
        axis.title=element_text(size=13,face="plain",color="black"),
        axis.text = element_text(size=12,face="plain",color="black"),
        legend.position="none"
  )


ggplot(mydata, aes(Class, TK))+
  geom_violin(aes(fill = Class),trim = FALSE)+
  geom_boxplot(width = 0.2)+
  scale_fill_manual(values=c(brewer.pal(7,"Set2")[c(1,2,4,5)]))+
  theme_classic()+
  theme(panel.background=element_rect(fill="white",colour="black",size=0.25),
        axis.line=element_line(colour="black",size=0.25),
        axis.title=element_text(size=13,face="plain",color="black"),
        axis.text = element_text(size=12,face="plain",color="black"),
        legend.position="none"
  )



ggplot(mydata, aes(Class, AP))+
  geom_violin(aes(fill = Class),trim = FALSE)+
  geom_boxplot(width = 0.2)+
  scale_fill_manual(values=c(brewer.pal(7,"Set2")[c(1,2,4,5)]))+
  theme_classic()+
  theme(panel.background=element_rect(fill="white",colour="black",size=0.25),
        axis.line=element_line(colour="black",size=0.25),
        axis.title=element_text(size=13,face="plain",color="black"),
        axis.text = element_text(size=12,face="plain",color="black"),
        legend.position="none"
  )



ggplot(mydata, aes(Class, AK))+
  geom_violin(aes(fill = Class),trim = FALSE)+
  geom_boxplot(width = 0.2)+
  scale_fill_manual(values=c(brewer.pal(7,"Set2")[c(1,2,4,5)]))+
  theme_classic()+
  theme(panel.background=element_rect(fill="white",colour="black",size=0.25),
```

```
        axis.line=element_line(colour="black",size=0.25),
        axis.title=element_text(size=13,face="plain",color="black"),
        axis.text = element_text(size=12,face="plain",color="black"),
        legend.position="none"
  )



ggplot(mydata, aes(Class, SOM))+
  geom_violin(aes(fill = Class),trim = FALSE)+
  geom_boxplot(width = 0.2)+
  scale_fill_manual(values=c(brewer.pal(7,"Set2")[c(1,2,4,5)]))+
  theme_classic()+
  theme(panel.background=element_rect(fill="white",colour="black",size=0.25),
        axis.line=element_line(colour="black",size=0.25),
        axis.title=element_text(size=13,face="plain",color="black"),
        axis.text = element_text(size=12,face="plain",color="black"),
        legend.position="none"
  )



ggplot(mydata, aes(Class, Hg))+
  geom_violin(aes(fill = Class),trim = FALSE)+
  geom_boxplot(width = 0.2)+
  scale_fill_manual(values=c(brewer.pal(7,"Set2")[c(1,2,4,5)]))+
  theme_classic()+
  theme(panel.background=element_rect(fill="white",colour="black",size=0.25),
        axis.line=element_line(colour="black",size=0.25),
        axis.title=element_text(size=13,face="plain",color="black"),
        axis.text = element_text(size=12,face="plain",color="black"),
        legend.position="none"
  )
```
