# Supplementary material for: High‐Altitude Open‐Pit Coal Mining has Changed the Sulfur Cycle and Ecological Network of Plant Rhizosphere Microorganisms
Source: Ecol Evol. 2025 Apr 11;15(4):e71183. doi: 10.1002/ece3.71183 (PMC11992355; doi:10.1002/ece3.71183)
Supplement: Supplementary file 5 — Data S2. [file ECE3-15-e71183-s004.docx]

**Appendix：**

**Table S1.** List of plant species in each type plots

| Plant name | Genera | Succession type |
| --- | --- | --- |
| *Oxytropis myriophylla* | *Oxytropis* DC. | MOR |
| *Agropyron cristatum* | *Agropyron* Gaertn. | MOR |
| *Saussurea pulvinata* Maxim | *Saussurea* DC. | MOR |
| *Ephedra monosperma* | *Ephedra* L. | MOR |
| *Artemisia austriaca* | *Artemisia* L. | MOR |
| *Elymus nutans* Griseb | *Elymus* Linn. | MRR |
| *Artemisia rupestris* | *Artemisia L.* | MRR |
| *Youngia heterophylla* | *Youngia Cass.* | MRR |
| *Stipa capillata* | *Stipa* | NMOR |
| *Chenopodium glaucum* | *Chenopodium* L. | NMOR |
| *Elymus nutans* Griseb | *Elymus* Linn. | NMOR |

**Table S2.** S cycle related microbial abundance, refer to the study by Yu et al (2021).

| Pathway | Domain | Phylum | Class | Order | Family | Genus | NMOR | MRR | MOR |
| --- | --- | --- | --- | --- | --- | --- | --- | --- | --- |
| SOX systems | Bacteria | Proteobacteria | Alphaproteobacteria | Rhizobiales | Rhodobacteraceae | *Bradyrhizobium* | 1.826120344 | 1.683435646 | 1.258559438 |
| SOX systems | Bacteria | Planctomycetes | Clostridia | Streptomycetales | Oxalobacteraceae | *Mesorhizobium* | 0.634172573 | 1.020030262 | 2.226339043 |
| Assimilatory sulfate reduction | Bacteria | Cyanobacteria | Betaproteobacteria | Bacillales | Mycobacteriaceae | *Streptomyces* | 0.824418834 | 0.185642284 | 0.210480219 |
| Assimilatory sulfate reduction | Bacteria | Thermus | Bacteroidia | Streptomycetales | Paenibacillaceae | *Burkholderia* | 0.072855368 | 0.146509548 | 0.653252572 |
| Assimilatory sulfate reduction | Bacteria | Deinococcus | Deltaproteobacteria | Alteromodales | Xanthomonadaceae | *Mycobacterium* | 0.246488383 | 0.209280944 | 0.284041896 |
| SOX systems | Bacteria | Chlorobi | Deinococci | Pseudomonadales | Phyllobacteriaceae | *Paracoccus* | 0.006543518 | 0.333076692 | 0.220091557 |
| Assimilatory sulfate reduction | Bacteria | Proteobacteria | Gammaproteobacteria | Enterobacterales | Enterobacteriaceae | *Pseudomonas* | 0.027078129 | 0.178420002 | 0.086196368 |
| Assimilatory sulfate reduction | Bacteria | Bacteroidetes | Bacilli | Vibrionales | Streptomycetaceae | *Escherichia* | 0.021017683 | 0.017453913 | 0.105691587 |
| SOX systems | Bacteria | Deinococcus Thermus | Epsilonproteobacteria | Campylobacterales | Enterobacteriaceae | *Methylobacterium* | 0.049938512 | 0.035935682 | 0.040276296 |
| Assimilatory sulfate reduction | Bacteria | Actinobacteria | Alphaproteobacteria | Pseudomodales | Vibrionaceae | *Vibrio* | 0.00809376 | 0.045119139 | 0.019227939 |
| Organic sulfur transformation | Bacteria | Tenericutes | Bacteroidia | Streptomycetales | Staphylococcaceae | *Acinetobacter* | 0.026590182 | 0.020817498 | 0.014493903 |
| SOX systems | Bacteria | Firmicutes | Gammaproteobacteria | Rhodobacterales | Burkholderiaceae | *Burkholderia* | 0.00903118 | 0.025914267 | 0.013723018 |
| SOX systems | Bacteria | Aquificae | Bacilli | Rhodospirillales | Campylobacteraceae | *Cupriavidus* | 0.00713407 | 0.030403155 | 0.01155673 |
| Organic sulfur transformation | Bacteria | Planctomycetes | Epsilonproteobacteria | Rhodobacterales | Rhizobiaceae | *Enterobacter* | 0.024954876 | 0.001286939 | 0.007239998 |
| Dissimilatory sulfur reduction and oxidation | Archaea | Thaumarchaeota | Halobacteria | Methanosarcinales | Nitrosopumilaceae | *Nitrosopumilus* | 0.003431446 | 0.001544182 | 0.003652906 |
| Linkages between inorganic and organic sulfur transformation | Archaea | Crenarchaeota | Methanococci | Methanobacteriales | Halobacteriaceae | *Methanosarcina* | 0.000974809 | 0.000277685 | 0.000140524 |
| Sulfur oxidation | Archaea | Candidatus Korarchaeota | Thermoplasmata | Nitrososphaerales | Halococcaceae | *Halobellus* | 8.96E-06 | 0.000612033 | 3.73E-05 |
| Sulfur reduction | Archaea | Candidatus Bathyarchaeota | Thermococci | Thermococcales | Candidatus Methanoperedenaceae | *Candidatus Methanoperedens* | 0.000116457 | 0.000160311 | 0.000138864 |
| SOX systems | Archaea | Euryarchaeota | Halobacteria | Haloferacales | Haloferacaceae | *Halorubrum* | 0.000126731 | 4.85E-05 | 0.000126728 |
| SOX systems | Archaea | Euryarchaeota | Methanococci | Methanococcales | Halobacteriaceae | *Haladaptatus* | 2.19E-05 | 1.37E-05 | 0.000121798 |
| Sulfur reduction | Archaea | Candidatus Bathyarchaeota | Thermoprotei | Haloferacales | Methanosarcinaceae | *Haloferax* | 6.49E-05 | 4.13E-05 | 6.35E-05 |
| Others | Archaea | Candidatus Korarchaeota | Thermoplasmata | Nitrososphaerales | Halococcaceae | *Halococcus* | 0.000114471 | 8.15E-06 | 3.31E-05 |
| Organic sulfur transformation | Archaea | Crenarchaeota | Methanomicrobia | Halobacteriales | Haloarculaceae | *Methanosarcina* | 0.00014335 | 0 | 1.13E-05 |
| Others | Archaea | Crenarchaeota | Archaeoglobi | Sulfolobales | Thermococcaceae | *Halorussus* | 0 | 0 | 8.41E-05 |
| Linkages between inorganic and organic sulfur transformation | Archaea | Candidatus Korarchaeota | Thermoplasmata | Nitrososphaerales | Halorubraceae | *Methanoculleus* | 5.08E-05 | 2.64E-05 | 2.28E-05 |
| Sulfur reduction | Archaea | Euryarchaeota | Methanomicrobia | Methanosarcinales | Thermococcaceae | *Pyrococcus* | 3.26E-05 | 2.26E-05 | 0 |
| Organic sulfur transformation | Archaea | Candidatus Korarchaeota | Thermococci | Thermoplasmatales | Methanoperedenaceae | *Methanolobus* | 0 | 0 | 2.38E-05 |

**Table S3.** The physichemical properties of rhizosphere soils of different succession types of plants

| Name | MRR | MOR | NMOR |
| --- | --- | --- | --- |
| Moisture content(%, SM) | 3.09±1.95a | 3.59±1.25a | 2.75±0.82a |
| Conductivity (ms/cm, Cond) | 0.24±0.13b | 0.73±0.30a | 0.11±0.029b |
| pH | 8.47±0.09a | 8.41±0.15a | 8.18±0.26a |
| SOM (g kg^-1^) | 11.73±4.09b | 20.36±3.48b | 50.52±13.04a |
| Total P (g kg^-1^, TP) | 0.51±0.01a | 0.32±0.06b | 0.76±0.14a |
| Available P (mg kg^-1^, AP) | 4.99±3.09b | 2.39±0.73c | 6.81±3.83a |
| Total N (mg kg^-1^, TN) | 0.52±0.14b | 0.83±0.15b | 2.55±0.69a |
| NH_4_^+^-N (mg kg^-1^, NH4) | 0.68±0.11c | 1.29±0.36b | 2.40±0.31a |
| NO_3_^-^-N (mg kg^-1^, NO3) | 3.55±0.15b | 12.73±7.45a | 9.14±2.23a |
| Total K(g kg^-1^, TK) | 19.66±0.06b | 20.75±0.11a | 19.36±0.45b |
| Available K(mg kg^-1^, AK) | 188.8±54.4b | 93.8±13.7b | 795.7±198.7a |

Note: Different lowercase letters indicate significant differences in rhizosphere soil in different regions.
